# Medical Students' Readiness for Prescription Communication Skills Training: A Needs Assessment Study

**DOI:** 10.7759/cureus.69013

**Published:** 2024-09-09

**Authors:** Padmanabha Thiruganahalli Shivaraju, Ravi Shankar Manchukonda, Tejaswi H Lokanathan, Haradanahalli G Kshamaa

**Affiliations:** 1 Pharmacology, PES University Institute of Medical Sciences and Research, Bengaluru, IND; 2 Pharmacology, Adichunchanagiri Institute of Medical Sciences, Mandya, IND; 3 Anatomy, Adichunchanagiri Institute of Medical Sciences, Mandya, IND; 4 Psychiatry, Kempegowda Institute of Medical Sciences, Bengaluru, IND

**Keywords:** communication skills, doctor-patient communication, medical students, needs assessment, negative attitude, positive attitude, prescription

## Abstract

Background and objectives

The quality of doctor-patient communication plays a crucial role in determining positive medical outcomes. Medical educators may be able to develop effective programs to orient the students toward learning communication skills with the aid of assessment of the attitude of medical students toward such learning. Recently, the National Medical Commission's updated syllabus strongly emphasized on the value of training in prescription communication skills (PCS), in pharmacology. Our study utilizes the Communication Skills Attitude Scale (CSAS) to explore medical students' attitudes toward learning PCS in a private medical college, aiming to address the dearth of data in the Indian context.

Methodology

This cross-sectional study assessed the attitudes of 131 second-year medical students at Adichunchanagiri Institute of Medical Sciences toward PCS training. Validated, 26-item CSAS was used to measure their attitudes which include 13 items of Positive Attitude Scale (PAS) and 13 items of Negative Attitude Scale (NAS), and data analysis was conducted using independent t-tests to explore potential associations based on socio-demographic factors.

Results

The study scale showed an acceptable internal consistency of 0.71 (Cronbach's alpha) with 0.92 and 0.76 for PAS and NAS, respectively. The overall mean PAS score and NAS score were 54.2±6.9 and 34.7±6.3, respectively, indicating that the majority of students recognized the significance of communication skills for their future medical practice. Male students had significantly lower PAS scores (52.1±7.4) compared to female students (55±6.6) (p=0.02). Students with a rural background had significantly higher PAS scores (56.2±6.1) (p=0.01) compared to those with an urban background (53.2±9.8). No significant association was seen with demographic parameters like schooling background, presence of doctors in the family, and mother tongue they spoke.

Conclusion

The study revealed that second-year medical students had a strong inclination toward learning PCS. Therefore, greater emphasis should be placed on providing adequate training in PCS to the students to ensure effective doctor-patient interactions.

## Introduction

In the domain of medical education, effective communication skills were once considered desirable attributes; however, they have now evolved into essential competencies in the current context.

The doctor-patient relationship, a cornerstone of healthcare delivery, hinges significantly on physicians' ability to communicate proficiently. Adept communication not only fosters treatment compliance and enhances health outcomes but also ensures higher satisfaction levels for both patients and healthcare providers. Active listening, empathy, and ethical awareness are vital components of effective communication, facilitating the gathering of pertinent information and the provision of emotional support to patients [1]. However, despite its paramount importance, deficiencies in communication skills among medical professionals have led to litigation and complaints, underscoring the need for comprehensive training in this area [2].

While medical trainees may acquire rudimentary communication skills through observational learning during internships, such skills often prove inadequate for navigating the complexities of patient interactions in their professional careers [3]. Notably, fifth-year medical students have exhibited significant deficits in effectively communicating drug prescriptions to patients, highlighting a critical gap in their training [4]. Addressing these deficiencies is crucial, given the pivotal role that effective communication plays in fostering optimal doctor-patient relationships and improving health outcomes. Furthermore, graduate medical education regulations underscore that effective communication is essential for medical professionals, emphasizing the need for structured communication skills training within medical curricula [5,6].

Students' preferences for learning communication skills vary widely [7]. Some may favor traditional methods, such as lectures, while others prefer experiential learning, including role-playing with simulated patients or interacting with real patients in clinical settings. Understanding these preferences is crucial for designing effective communication training programs tailored to students' needs. Additionally, factors such as gender, parental background, and perceived areas for improvement influence attitudes toward communication skills acquisition [8]. The Communication Skills Attitude Scale (CSAS), developed by Rees et al., is a well-established tool for assessing attitudes toward learning communication skills. It enables researchers to explore how medical students' attitudes are influenced by factors such as their demographic and educational backgrounds [8].

This article aims to explore undergraduate medical students' readiness to acquire prescription communication skills (PCS), addressing a notable gap in existing research and aiding the development of more effective communication training strategies tailored to meet the needs of aspiring healthcare professionals.

The primary objective of this study was to assess the needs related to undergraduate medical students' positive attitudes and negative attitudes toward acquiring PCS and to correlate their CSAS with demographic variables such as gender, language, schooling, and family backgrounds.

## Materials and methods

Study design

This was a cross-sectional study conducted in July 2022 to assess the attitude toward PCS training among second-year medical students at the Adichunchanagiri Institute of Medical Sciences, Mandya.

Study population and sampling

A total of 131 second-year undergraduate medical students were enrolled in the study using convenience sampling.

Inclusion criteria

Participants who voluntarily agreed to participate and provided written informed consent were included in the study.

Exclusion criteria

Students who did not complete the questionnaire or provided incomplete responses were excluded from the study.

Ethical approval

The study was approved by the Institutional Ethics Committee of Adichunchanagiri Institute of Medical Sciences (approval number AIMS/IEC/020/2022, issued on June 11, 2022).

Study tool

Participants' attitudes toward communication skills training were assessed using the validated CSAS, which consists of 26 questions [8]. The CSAS includes 13 questions related to the Positive Attitude Scale (PAS) and 13 questions related to the Negative Attitude Scale (NAS) toward communication skills training. Permission to use the CSAS was obtained from Rees et al., the developers of the scale [8]. Each question is scored from 1 (strongly disagree) to 5 (strongly agree), with higher scores indicating stronger attitudes. The CSAS has demonstrated satisfactory internal consistency and test-retest reliability [8]. Two additional questions were included to assess participants' current level of PCS in practice as students, using a 5-point scale from 0 (very poor) to 4 (very good), and to evaluate the perceived need for PCS improvement.

Data collection procedure

The demographic details of the study participants, such as gender, residence, type of school syllabus studied, presence or absence of doctors in the family, and the language spoken at home (Kannada or other languages), were collected. Participants were first sensitized to the use of the CSAS during a 15-minute session. All items in the questionnaire were of the closed-ended type. Responses were collected electronically using Google Forms, and participants completed the CSAS questionnaire within 30 minutes, including questions about current levels of PCS and the need for further training to improve PCS. Participants' anonymity and confidentiality were maintained throughout the study.

Statistical analysis

Descriptive statistics, including mean, standard deviation, and percentages, were calculated. Data analysis was performed using IBM SPSS Statistics for Windows, Version 26.0 (Released 2019; IBM Corp., Armonk, New York, United States) and Microsoft Excel. Independent t-tests were conducted to explore potential associations between participants' attitudes toward communication skills training and demographic variables such as gender, language, and schooling background. A p-value of less than 0.05 was considered statistically significant.

## Results

Among 131 students, 94 (71.8%) were females, while the rest were males. CSAS showed that students had a positive attitude toward learning PCS, with a mean score of 54.2±6.9 compared to a mean of 34.7±6.3 in NAS (Table 1). The difference between the mean scores of PAS and NAS was statistically significant (p=0.0001). There were statistically significant differences in PAS scores for gender and residence; female students and those from rural backgrounds had higher PAS scores than their counterparts (Table 1). No significant differences were observed in relation to other demographic variables for either CSAS-PAS or CSAS-NAS (Table 1).

**Table 1:** Demographic comparison of PAS scores and NAS scores among medical students PAS: Positive Attitude Scale; NAS: Negative Attitude Scale; SD: standard deviation; ICSE: Indian Certificate of Secondary Education; CBSE: Central Board of Secondary Education *The difference between PAS and NAS scores was statistically significant (p=0.0001).

Demographic	Categories	Frequency (n=131)	PAS (mean±SD)	P-value	NAS (mean±SD)	P-value
Gender	Male	37	52.1±7.4	0.02	37.2±6.6	0.17
	Female	94	55±6.6		33.8±5.9	
Residence	Rural	44	56.2±6.1	0.01	36.5±6	0.32
	Urban	87	53.2±9.8		33.8±12.1	
Syllabus	State	56	54.5±9.1	0.58	35.75±7.1	0.46
	ICSE/CBSE	75	53.9±7.1		33.9±5.4	
Doctors in the family	Yes	94	54±6.6	0.49	34.7±6.1	0.96
	No	37	54.7±7.8		34.8±6.2	
Language spoken	Kannada	108	54.4±6.9	0.41	34.5±6.3	0.7
	Others	23	53.1±6.6		35.4±6.3	
Total*	Students	131	54.2±6.9	-	34.7±6.3	-

Table 2 and Table 3 highlight the mean scores of students' positive attitudes and negative attitudes toward PCS, respectively. 

**Table 2:** Assessment of PAS scores among medical students PAS: Positive Attitude Scale; SD: standard deviation

No.	Questions of PAS	Mean	SD
1	In order to be a good doctor, I must have good communication skills	4.57	0.73
4	Developing my communication skills is just as important as developing my knowledge of medicine	4.39	0.77
5	Learning communication skills has helped or will help me respect patients	4.37	0.69
7	Learning communication skills is interesting	4.01	0.73
9	Learning communication skills has helped or will help facilitate my team-working skills	4.24	0.71
10	Learning communication skills has improved my ability to communicate with patients	4.23	0.73
12	Learning communication skills is fun	3.80	0.83
14	Learning communication skills has helped or will help me respect my colleagues	4.18	0.65
16	Learning communication skills has helped or will help me recognize patients' rights regarding confidentiality and informed consent	4.15	0.68
18	When applying for medicine, I thought it was a really good idea to learn communication skills	3.89	0.78
21	I think it's really useful learning communication skills on the medical degree	4.20	0.72
23	Learning communication skills is applicable to learning medicine	3.92	0.78
25	Learning communication skills is important because my ability to communicate is a lifelong skill	4.28	0.67

**Table 3:** Assessment of NAS scores among medical students NAS: Negative Attitude Scale; SD: standard deviation

No.	Questions of NAS	Mean	SD
2	I can't see the point in learning communication skills	4.54	0.61
3	Nobody is going to fail their medical degree for having poor communication skills	4.24	0.86
6	I haven't got time to learn communication skills	4.14	0.82
8	I can't be bothered to turn up to sessions on communication skills	3.86	0.95
11	Communication skills teaching states the obvious and then complicates it	4.08	0.72
13	Learning communication skills is too easy	4.11	0.77
15	I find it difficult to trust information about communication skills given to me by non-clinical lecturers	3.51	1.02
17	Communication skills teaching would have a better image if it sounded more like a science subject	4.03	0.76
19	I don't need good communication skills to be a doctor	4.00	0.71
20	I find it hard to admit to having some problems with my communication skills	3.76	0.86
22	My ability to pass exams will get me through medical school rather than my ability to communicate	4.00	0.75
24	I find it difficult to take communication skills learning seriously	3.78	0.82
26	Communication skills learning should be left to psychology students, not medical students	4.14	0.71

**Figure 1:**
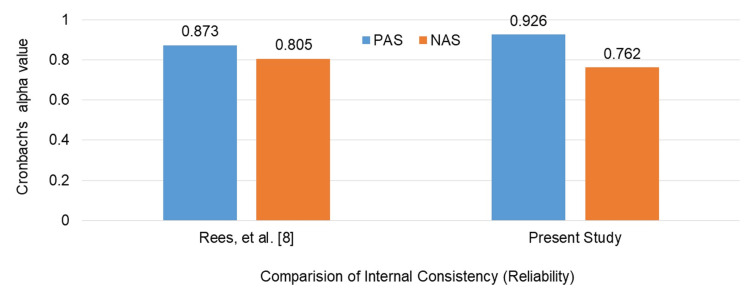
Comparison of internal consistency (Cronbach's alpha) of CSAS CSAS: Communication Skills Attitude Scale

The present study showed internal consistency for the CSAS comparable to that reported by Rees et al. with a Cronbach's alpha value of 0.926 for the PAS and 0.762 for the NAS (Figure 1). 

**Figure 2:**
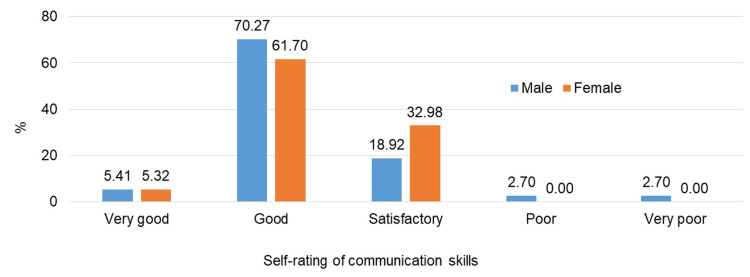
Self-rating of students' communication skills on a Likert scale %: percentage

A total of 70.27% of female students and 61.70% of male students self-rated that their PCS are good. Only a few students, 5.32% of females and 5.41% of males, perceived their communication skills to be very good (Figure 2).

**Figure 3:**
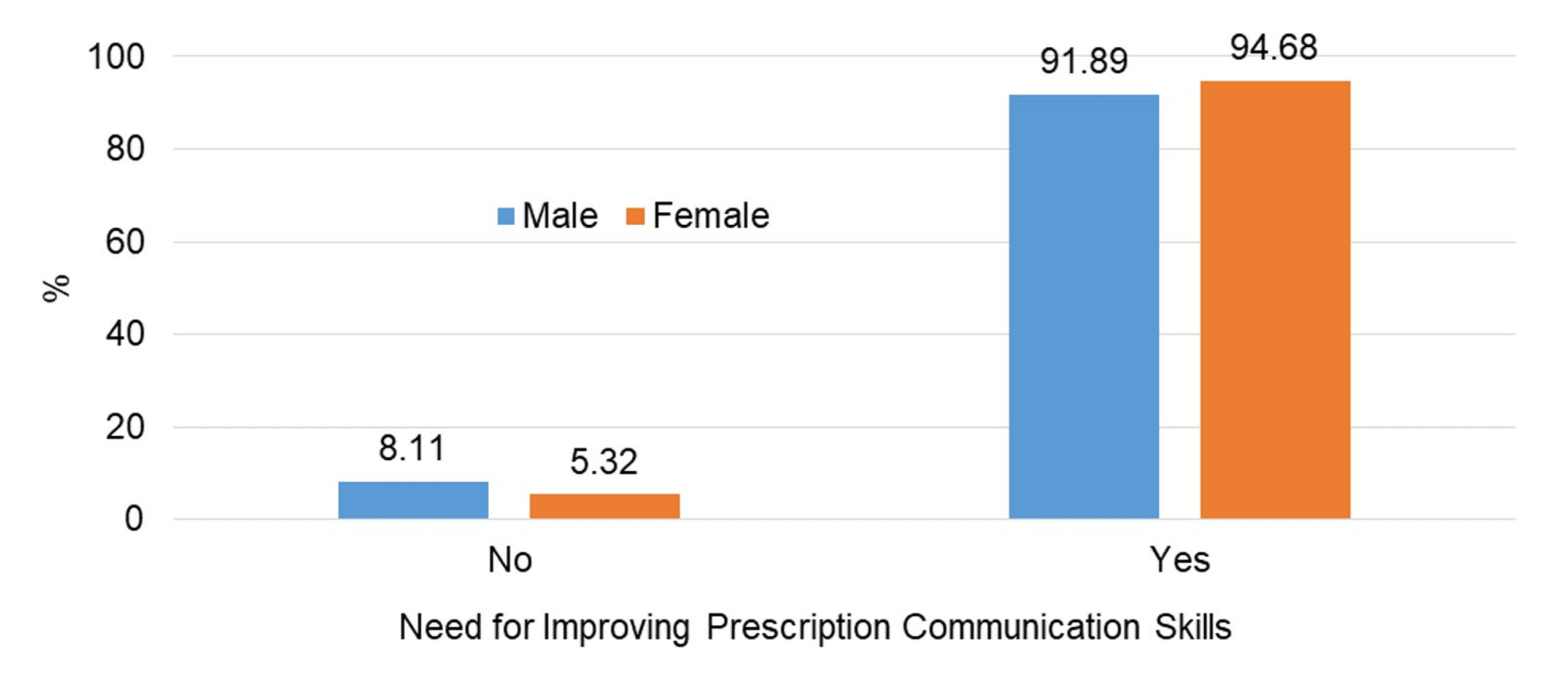
Students' perceived need for communication skills improvement %: percentage

The majority of students, both male (91.89%) and female (94.68%), expressed a desire for further improvement in their PCS through structured training (Figure 3).

## Discussion

The results of our study indicate that the PAS and the NAS exhibited excellent and acceptable internal consistency, respectively (Figure 1), consistent with earlier studies [8,9]. This indicates that the measurement tools employed in our research are both reliable and aligned with established standards in the field.

This cross-sectional study, the first of its kind among 131 second-year medical undergraduates, focused on evaluating readiness to learn PCS in India. Our findings revealed an overall positive attitude (mean PAS: 54.2±6.9) (Table 1) toward learning communication skills, which was statistically significant (p=0.0001) (Tables 1-3), aligning with prior research on the subject [10-12].

Consistent with existing literature, we observed gender disparities in attitudes toward communication skills learning, with female students demonstrating higher positive attitudes (p=0.02) and lower negative attitudes compared to their male counterparts (Table 1) [12-18]. In contrast, a study by Shankar et al. found no gender differences [19]. These findings suggest that female students may exhibit more partnership-building behavior, potentially due to socialization processes and a generally more empathic nature.

Additionally, while previous research has linked having parents who are doctors or nurses to higher negative attitudes toward communication skills learning [13], our study did not find any significant variation regarding the influence of having doctors in the family on attitudes. This may suggest a shift or variation within this demographic, emphasizing that individual communication skills are more likely influenced by personal motivation and a desire to self-reflect rather than by a parent's profession.

Contrary to previous findings, where students speaking the local language had more positive attitudes than others, our study showed that language proficiency did not significantly impact students' attitudes (Table 1) [14]. We also found no significant differences in attitudes based on students' schooling backgrounds. This discrepancy highlights the importance of contextual factors in shaping attitudes toward communication skills learning and emphasizes the need for further investigation into cultural and linguistic influences on educational perceptions. However, students from rural backgrounds had statistically significantly better communication abilities for PAS than those from urban backgrounds (p=0.01) (Table 1), which may be influenced by social interactions at home.

Students in this study had higher mean scores for positive attitudes (PAS: 54.2±6.9) (Table 2) compared to negative attitudes (NAS: 34.7±6.3) (Table 3), indicating a general consensus that to become a good doctor, one must excel at communication, which is crucial in patient care. The higher positive attitude among students would also help the facilitator frame the curriculum based on students' motivation [12]. However, the presence of higher scores for negative attitudes highlights the need for immediate attention to the importance of learning communication skills. Changing these attitudes and emphasizing the importance of improving communication in daily practice can significantly impact time management and patient care quality.

A majority of the students self-rated their PCS as good or satisfactory (Figure 2), which can be attributed to individual confidence, self-motivation, interaction abilities, and active participation. Additionally, a large proportion of students showed a keen interest in improving their PCS (Figure 3). This finding is consistent with studies conducted at the School of Medicine of the Universitat Autònoma de Barcelona in 2011 [14] and in Pokhara, Nepal [19], indicating the necessity of incorporating PCS into the current training program. This aligns with the competency-based medical education curriculum provided by the National Medical Commission [5].

Overall, our study provides valuable insights into the attitudes of medical undergraduates toward communication skills learning, confirming the reliability of the measurement tools and shedding light on potential demographic and cultural factors influencing attitudes. Future research should continue to explore these factors to inform the development of targeted interventions aimed at optimizing communication skills training within medical education.

Strengths and limitations

The study adapted and validated the Indian version of the CSAS through internal consistency analysis among medical students. Our study conclusions are based on data from a single center and one batch of medical students, which may limit the generalizability of the findings. The results of this study open opportunities for further research involving larger cohorts and multicenter studies. Further validation of the Indian version of the CSAS among healthcare professionals, including students, would enhance its usefulness in training for PCS.

## Conclusions

This study explored medical students' attitudes toward acquiring PCS and examined their correlation with demographic variables. Overall, students exhibited positive attitudes toward learning communication skills, with notable differences based on gender. However, no significant variations were observed concerning language, schooling, or family background. These findings underscore the need for tailored interventions to enhance communication skills training in medical education, ultimately improving patient care. Further investigation into influencing factors will be crucial for developing targeted educational strategies.
